# The Impact of Antenatal Psychological Group Interventions on Psychological Well-Being: A Systematic Review of the Qualitative and Quantitative Evidence

**DOI:** 10.3390/healthcare4020032

**Published:** 2016-06-08

**Authors:** Franziska Wadephul, Catriona Jones, Julie Jomeen

**Affiliations:** Faculty of Health and Social Care, University of Hull, Hull HU6 7RX, UK; c.jones@hull.ac.uk (C.J.); j.jomeen@hull.ac.uk (J.J.)

**Keywords:** perinatal mental health, antenatal intervention, antenatal depression, postnatal depression, antenatal anxiety, antenatal stress, systematic review

## Abstract

Depression, anxiety and stress in the perinatal period can have serious, long-term consequences for women, their babies and their families. Over the last two decades, an increasing number of group interventions with a psychological approach have been developed to improve the psychological well-being of pregnant women. This systematic review examines interventions targeting women with elevated symptoms of, or at risk of developing, perinatal mental health problems, with the aim of understanding the successful and unsuccessful features of these interventions. We systematically searched online databases to retrieve qualitative and quantitative studies on psychological antenatal group interventions. A total number of 19 papers describing 15 studies were identified; these included interventions based on cognitive behavioural therapy, interpersonal therapy and mindfulness. Quantitative findings suggested beneficial effects in some studies, particularly for women with high baseline symptoms. However, overall there is insufficient quantitative evidence to make a general recommendation for antenatal group interventions. Qualitative findings suggest that women and their partners experience these interventions positively in terms of psychological wellbeing and providing reassurance of their ‘normality’. This review suggests that there are some benefits to attending group interventions, but further research is required to fully understand their successful and unsuccessful features.

## 1. Introduction

### 1.1. Background

Maternal antenatal depressive and anxiety disorders are a major issue for many women, not only affecting neonatal outcomes at birth [1,2,3], but having long term effects on behavioural and cognitive outcomes for children [4,5,6,7]. Antenatal anxiety and depression are prevalent and serious problems with changing courses [8]. The prevalence of moderate to high levels of depressive symptoms during pregnancy varies by population, screening instruments, criteria used and timing of assessment [9]. Whilst figures suggest that approximately 13% of women experience postnatal depression (PND), this is matched by that of antenatal depression [10,11], and antenatal depressive and anxiety disorders have become increasingly recognised as conditions in their own right and significant issues for many pregnant women [12,13]. While perinatal mental illness (PMI) encompasses a range of mental health conditions, this review focuses on anxiety, depression and stress, as these are the most prevalent conditions during the antenatal period.

Research has shown that perinatal vulnerability to PND begins before birth [11,14,15]. There is considerable literature on risk factors and neonatal and obstetric outcomes for perinatal mental illness (PMI). In view of its prevalence and potentially significant consequences, there has been growing interest in identifying antenatal interventions which facilitate the process of preventing or managing PMI and/or optimising psychological well-being before and after women give birth. These antenatal interventions vary widely in their approach, including antenatal education, physical activities, and interventions with a psychological focus. The focus of this review will be on interventions which use a psychological approach with a clear theoretical underpinning and evidence of effectiveness in other clinical areas. More specifically, the focus will be on group interventions, as these have pragmatic advantages and the potential to reach a large number of women, as well as providing an element of peer support, which an increasing body of evidence suggests has some demonstrable benefits [16,17,18,19,20,21].

Despite the increasing use of antenatal group interventions significant gaps in the research remain with respect to their effectiveness in the treatment of maternal antenatal depressive and anxiety disorders. This systematic review aims to draw together the evidence from quantitative and qualitative studies evaluating the efficacy of psychologically-focused antenatal group interventions targeting women with elevated symptoms of depression, anxiety and/or stress or who have an increased risk of perinatal mental illness.

### 1.2. The Theoretical Bases of Interventions

While this review was not limited to specific psychological interventions at the outset, the interventions in this review were largely based on three approaches: Cognitive-behavioural therapy (CBT), interpersonal therapy (IPT) and mindfulness (MFN). For clarity these approaches will briefly be described here. Cognitive behavioural therapy (CBT) is widely used to treat anxiety and depression, including in a perinatal context [22,23]. It is based on the theory that thoughts, emotions and behaviours are interconnected: Distorted, negative thinking affects feelings and behaviours and can lead to negative patterns. The focus in CBT is on changing thoughts and behaviours in order to manage problems in a more positive, constructive way. This involves breaking problems down into smaller parts and acquiring coping strategies and skills. The focus is on current issues rather than the past. In pregnancy, these distorted thoughts may relate to issues around pregnancy, labour and early parenting; addressing these pregnancy-specific negative thoughts may be particularly beneficial.

Interpersonal therapy is a time-limited, structured form of therapy which relates psychological distress to difficulties in interpersonal relationships [24]. IPT focuses not just on psychological symptoms, but also on conflicts in relationships and social support. IPT may be particularly suitable to the perinatal context as this involves fundamental changes in interpersonal relationships, the role transition to motherhood and an increased need for social support. There is a wide evidence base for IPT, including its use during the perinatal period [24,25,26,27].

Mindfulness-based approaches derive from Buddhist traditions and aim to cultivate “a mental state of awareness and acceptance of present moment experiences, including one’s current sensations, thoughts, bodily states and environment” [28]. They use techniques such as progressive muscle relaxation, yoga and various forms of meditation and are often taught in groups. Mindfulness-based stress reduction programs [29] have been found to be successful in reducing anxiety and psychological distress and improving health outcomes [30,31]. Mindfulness-based interventions aim to provide strategies for managing negative emotions and stress and tend to focus on the reduction of anxiety and stress.

## 2. Materials and Methods

### 2.1. Search Strategy

**Figure 1:**
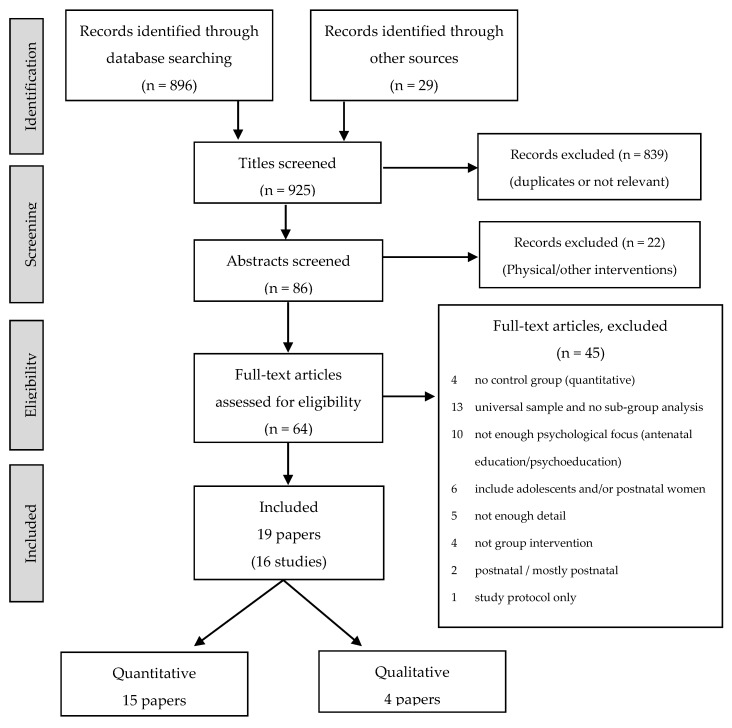
PRISMA flowchart of review phases, adapted from Moher *et al.* [34].

Electronic searches using Medline, PsycINFO and CINAHL were carried out using the search terms “group intervention”, “anxiety”, “depression”, “stress”, “pregnancy”, “antenatal” and “prenatal”; “qualitative” was used in addition to search for qualitative papers (Figure 1). Reference lists of identified papers were searched for relevant publications and selected journals were hand searched.

### 2.2. Inclusion and Exclusion Criteria

Included were qualitative studies and quantitative studies using a control group which evaluated interventions for the prevention and treatment of PMI. Studies were only included if they either included participants with elevated baseline symptoms and/or at high risk of PMI, or used a universal sample but provided a subgroup analysis of women with elevated baseline symptoms. Studies with adolescents were excluded, as were those which focused on participants with specific problems, such as tocophobia or premature birth. Only interventions which had taken place predominantly in a group setting and during pregnancy and which used recognised, theory-based psychological approaches were included. To be included, studies needed to assess the impact on psychological well-being, with a focus on depression, anxiety and stress as these potentially have significant adverse effects on maternal and child well-being and are the most prevalent mental health conditions in pregnancy.

### 2.3. Data Extraction

For each quantitative study relevant data on study design, type of intervention, control, participants and outcome measures were entered into a specially designed form and then summarised in a table. Intervention formats and study design, including participant characteristics, were summarised for each study to facilitate comparisons. The effectiveness of each intervention in terms of reducing or preventing depression, anxiety and/or stress was assessed in terms of the impact of participant and intervention characteristics. Comparisons were drawn between interventions using different psychological interventions, as well as with those using the same approach. An early decision was made not to conduct a meta-analysis due to the evident diversity of outcome measures and study design, as well as methodological limitations within a number of studies.

For the qualitative studies details of study design, type of intervention and participants and a brief summary of findings were extracted into a table. The synthesis of qualitative studies was guided by Thomas and Harden (2008) [32], who propose a three-stage approach: Coding of the text “line-by-line”; the development of “descriptive themes”; and the generation of “analytical themes”. The authors originally planned to extract and synthesise study findings according to a broad review question of “*What are women’s experiences of antenatal psychological group interventions?*” Thomas and Harden suggest allowing for the possibility that a modified framework for stage 1 may be a better fit, as opposed to the use of a priori framework implied by the review question onto study findings. Being guided by Thomas and Harden the review question was temporarily placed aside, and thematic synthesis was conducted from the study findings themselves. During stage 3, the generation of analytical themes, the authors returned to the review question, to ensure that the synthesis product addressed directly the concerns of the review.

### 2.4. Quality Assessment

For the quantitative studies, study quality, particularly risk of biases, was assessed using a form based on the NICE guidelines manual [33] with added categories specific to this review. The qualitative studies were assessed using Critical Appraisal Skills Programme (CASP) guidelines. Quantitative and qualitative studies were assessed by one author each (FW and CJ, respectively), with all authors assessing a selection of papers. There was broad agreement with decisions made about the quality of studies and each study was assigned a category of high (A), medium (B) and low quality (C) (Table 1).

## 3. Results

### 3.1. Identified Studies

The systematic review identified 19 papers describing a total of 15 studies. Fifteen quantitative papers met inclusion criteria; two describe the same study with different outcome measures [35,36]. Four papers describing 3 qualitative studies were identified. Three papers, two qualitative [37,38] and one quantitative [39], refer to the same study. Details of the interventions, participants and studies are shown in Table 1.

### 3.2. Interventions

#### 3.2.1. Psychological Approaches

The psychological approaches used in the interventions fell broadly into three categories: CBT, IPT and MFN, though some interventions used a combination of these approaches. Table 1 provides further details of intervention aims, approaches and formats.

#### 3.2.2. Intervention Formats

The majority of CBT- and MFN-based interventions included 6 to 8 sessions, usually weekly, lasting 90 min or 2 h. The IPT-based interventions were noticeably shorter, consisting of four sessions of either 1 h or 90 min; the total number of hours ranged from 4 to 24 (Table 1). Several interventions included additional elements, such as postnatal booster sessions.

Group size ranged from three to five women to 20. Not all studies gave information about the size of groups and often group size was not determined at the start of the study but depended on how many women were recruited at the same time. High attrition in some studies led to some groups being very small. Partners were invited to take part in only one of the interventions [40]; in another [30,37,38] partners were invited to attend the session focusing on PND.

The interventions were facilitated by a variety of people, ranging from clinical psychologists to nurses and occupational therapists (Table 1). The training and supervision given to facilitators also seemed to vary and it is conceivable that in some cases training may have been rather brief. Most of the interventions were checked for fidelity or evaluated in some way (Table 1), either through participant feedback or by audio or video taping and then subsequently reviewing sessions.

### 3.3. Study and Participant Characteristics

#### 3.3.1. Study Characteristics

While some of the studies were small pilot studies, others were intended to be larger but had considerable problems with recruitment and/or attrition (Table 1). Non-completion of data was a serious problem for some studies, particularly the CBT-based interventions. Participation in interventions also varied and was low for all CBT-based interventions. While it is conceivable that a large number of women may have simply lost interest, there were also specific reasons why women dropped out, such as time constraints [23,28,35,41], lack of childcare [23,28], transport problems [23,28,42], health problems [28,42], giving birth [43] and moving out of the area [44]. In one study cultural reasons may have accounted for the high attrition before the intervention started: women did not want to say no even if they had no intention of taking part [42]. There was speculation that women with symptoms which were just above the baseline threshold were not motivated enough to take part as they did not see the need for the course [23,35,36].

There was considerable heterogeneity in terms of the focus, method and timing of screening (Table 1). Most studies screened for elevated depression and anxiety symptoms and/or increased risk due to personal or family history. The proportion of women who screened positive was not given by all studies, but the considerable variation indicates that screening criteria differed considerably between studies. The diverse approaches to screening make it difficult to compare the studies in terms of participants’ psychological baseline status.

The majority of the quantitative studies used “care as usual” as a control. Two studies [23,28] used a reading control group; another study [45] used two control groups: One receiving usual care and another with women at low risk of depression. A further study [46] used a wait-list control, with the control group receiving the same intervention postnatally and only one study [40] used a routine antenatal course as a control.

The most frequent measures in the quantitative studies were the Edinburgh Postnatal Depression Scale (EPDS) [47], Center for Epidemiologic Studies Depression (CES-D) scale [48] and the Beck Depression Inventory (BDI) [49] for depressive symptoms, the State-Trait Anxiety Inventory (STAI) [50] for anxiety and the Perceived Stress Scale (PSS) [51] for stress (Table 1). The EPDS was originally developed for screening for postnatal depression [47], but has been widely used and validated for pregnant populations [52,53]. While the CES-D has been used extensively in pregnancy, the evidence for its validity in pregnancy is less robust than for the BDI [52,54], the STAI [55] and the PSS [56].

All studies took baseline measurements before the intervention and most made further assessments immediately after the intervention and at one or more postnatal time point. As many studies included women with a wide range of gestational ages, the length of time after women had given birth varied considerably at the postnatal timepoints. This may have affected outcome measures, as psychological well-being is likely to have been influenced by the timing after birth [57]. The two quantitative MFN studies [28,46] took measurements at a specific time post-intervention; it was not clear whether all women had given birth at this point.

#### 3.3.2. Participant Characteristics

All interventions targeted women with impaired psychological well-being or at risk of impaired psychological well-being, except two studies [40,41], which used a universal sample but provided a subgroup analysis for women with elevated baseline depressive symptoms. Bittner *et al.* [29] and Richter *et al.* [30] intended to target women with elevated depressive, anxiety and stress symptoms, but most of the women in their sample were within the normal range; Bittner *et al.* [35] completed a subgroup analysis of women with increased symptoms.

Table 1 gives details of the participants’ demographic characteristics. Most of the participants fall into two categories: (1) women who are mostly white, married/partnered, slightly older and with a medium-high income [23,28,35,36,40,46,58] and (2) women who are mostly African-American and/or Hispanic, slightly younger and with a low income [43,44,45,59,60,61,62].

Most women were in the second or third trimester of pregnancy. Information on gestational age was only provided in some studies (Table 1) and only two studies stated that the intervention was targeted at a specific gestational age: Over 28 weeks [37,38,39] and over 25 weeks [40]. In other studies gestational ages of participants appeared to depend largely on how many women were recruited at the same time, resulting in a wide range of gestational ages in some cases, e.g., 6 to 27 weeks at the start of the intervention [45].

### 3.4. Quantitative Studies: Findings

There was considerable heterogeneity in outcome measurements in terms of what was measured and how and when it was measured. The majority of studies assessed depressive symptoms and/or depressive episodes. Anxiety was measured by five studies and stress by six studies. Some secondary outcomes relevant to psychological well-being in the perinatal period were also measured by some studies. Statistically significant outcomes are shown in Table 1.

#### 3.4.1. Depressive Symptoms

The effect on depressive symptoms was assessed in twelve studies, but only two, both CBT-based, found some evidence of effect. Le and colleagues [60] found significantly lower depressive symptoms immediately post-intervention in the intervention group than the control group; this effect was not sustained postnatally. These results may need to be treated with caution as this study was assessed as being of poor quality, largely due to the high attrition and low participation rates: Almost 45% of women attended fewer than half of the eight sessions. As no data is provided for either non-attenders or participants who dropped out, it is impossible to know if there were differences between the groups. While Bittner and colleagues [35] found no overall significant differences between groups, a subgroup analysis of women with baseline EPDS scores ≥ 10 showed a significant decline in depressive symptoms for the intervention group compared to the control group 3 months postnatally. However, these results are based on only eleven women and are hence underpowered.

#### 3.4.2. Depressive Episodes

The effect on the incidence of depressive episodes was measured in ten studies, four of which found evidence of a decrease after the intervention. Lara and colleagues [42] found that the incidence of new major depressive episodes was significantly lower in the intervention group at 6 weeks and 4–6 months postnatally, though no intervention effect on depressive symptoms was found. One intervention [40] had a significant mitigating effect on the prevalence of postnatal depression for women with antenatal depression (Leverton Questionnaire score ≥ 12), resulting in an absolute risk reduction of 17.8% at 6 to 8 weeks postnatally. Two studies evaluating IPT-based interventions [44,62] also found a significantly lower risk of developing PND in the intervention group; however, these studies are of relatively poor quality (see Table 1) and results must therefore be treated cautiously.

#### 3.4.3. Anxiety

The effect of interventions on anxiety was assessed for three CBT-based interventions and the two MFN-based interventions [28,46]. The latter, which are of poor quality (Table 1), found evidence of an effect on pregnancy-specific anxiety [28] and state anxiety [46] immediately after the intervention; these effects were not sustained at the later time points.

#### 3.4.4. Stress

Stress was assessed by six studies using CBT, IPT and MFN-based approaches . While Leung and Lam [41] found that perceived stress was reduced immediately after an IPT-based intervention, Urizar and Muñoz [45] found that perceived stress had increased postnatally after a CBT-based intervention; however, the latter assessed perceived stress with one question asking participants to rate their stress levels from 1 to 100, which may have been less valid than the PSS used by Leung and Lam [41]. Two studies exploring CBT-based interventions assessed the biological stress response via salivary cortisol levels. Cortisol, which is secreted after activation of the hypothalamic-pituitary-adrenal axis during stress, has been extensively used as a biomarker for stress [63], with higher levels of cortisol indicating increased stress levels. The diurnal pattern is also of importance, with a steeper decrease of cortisol from morning to evening indicating a more normal stress response [45]. Both studies measured diurnal salivary cortisol in the morning and evening once at each time point. Richter *et al.* [36] found reduced levels, *i.e.*, an improvement in the biological stress response, post-intervention. Urizar and Muñoz [45] found reduced maternal cortisol levels 18 months after birth and reduced infant cortisol levels 6 months after birth. The studies assessing MFN-based interventions did not demonstrate a significant effect on stress levels [28,46].

#### 3.4.5. Potential Negative Effects of Interventions

Two studies suggested that intervention may have had negative effects, though other possible negative effects cannot be excluded as not all studies reported complete results. Urizar and Muñoz [45] found some evidence that 6 and 18 months after the birth women who attended more sessions reported significantly higher perceived stress levels than those who had attended fewer classes. The authors speculate that this may have been due to increased awareness of how critical the postnatal period is to infant development. Women in this group also showed reduced negative affect, which may indicate that even though they may have had higher stress levels, they were better able to regulate negative mood. The study by Kozinszky and colleagues [40] suggests that single women and women who had reported financial difficulties had an increased risk of PND after the intervention compared to women in the control group, possibly due to increased awareness of these problems and a comparison with women in the group whom they perceived as better off.

#### 3.4.6. Impact of Participant Characteristics

The demographic and obstetric characteristics of participants did not seem to affect outcomes, but there is some evidence that women with higher baseline symptoms benefit more from some interventions than women with low baseline symptoms. Bittner and colleagues [35] found that while there was no evidence of an overall effect of the intervention on depressive symptoms, there was a significant reduction in depressive symptoms in the intervention group for women with EPDS scores ≥ 12. Another study [40] found an absolute risk reduction of almost 18% in women diagnosed with antenatal depression, but only 0.4% for those without antenatal depression. There was some evidence of increased effectiveness of the intervention for women with high baseline symptoms of depression [42,60] and anxiety [42], though these differences did not reach statistical significance. A subgroup analysis of women with EPDS scores ≥ 12 by Leung and Lam [41] found that the intervention may have additional benefits in stress reduction.

#### 3.4.7. Impact of Intervention Characteristics

There is no consistent evidence that one of the three psychological approaches is more successful. CBT- and IPT-based interventions were most successful in reducing depressive symptoms and the occurrence of depressive episodes; however, many studies CBT- and IPT-based interventions showed no effect. The two mindfulness-based interventions were the only ones to have a significant impact on anxiety levels. However, these interventions had a stronger focus on anxiety symptoms than the majority of CBT- and IPT-based interventions, only three of which measured impact on anxiety. Furthermore, these two studies were small and of relatively poor quality and results should, therefore, be treated with caution.

While the format of interventions differs considerably in terms of length, group size, type of facilitator and inclusion of other elements, there was no discernible pattern of how these factors may have affected outcomes. Only one intervention included partners in all sessions [40]. While this intervention had some success in reducing the incidence of postnatal depression in women who were depressed during pregnancy, it is not possible to draw conclusions from this one case. However, the inclusion of partners may be particularly relevant for IPT-based interventions which have a strong focus on relationships and support.

Several studies reported on the differences between attenders and non-attenders or high-attenders and low-attenders. While most did not find any evidence that those who attended more sessions benefitted more [28,39,46], Le and colleagues [60] found that higher levels of participation increase the size of effects but did not change significance levels considerably. However, Urizar and Muñoz [45] found that higher attendance had some significant effects: Increased perceived stress levels postnatally, less negative affect 6 months postnatally, lower maternal morning cortisol levels postnatally and lower evening cortisol levels in infants.

### 3.5. Findings from the Thematic Synthesis of Qualitative Studies

#### 3.5.1. Qualitative Approaches

Methods of data collection and analysis in the qualitative studies varied. Sample sizes ranged from 9 to 39, with a total of 72 pregnant women in all three studies. The studies described and illustrated aspects of the experiences of pregnant women engaging with group based psychological interventions. Data collection methods included written completion of open-ended questions [58] and interviews [37,38,61]. Qualitative data were analysed using grounded theory [37,38], inductive comparative analysis [61] and qualitative content analysis [58].

#### 3.5.2. Analytical Themes

The data suggests that group-based interventions with a psychological approach for targeted women provide a supportive mechanism for women to move from fear and anxiety about the unknown to, not only a healthy acceptance of their fears and anxieties, but a new-found confident and empowered sense of self. The qualitative synthesis process produced four themes relating to the impact and experience of group based interventions. These were “connecting”, “sharing”, “understanding” and “re-adjusting”.

##### Connecting

Connecting with other participants and developing supportive friendships was identified as an important aspect of the experience. Women valued opportunities to meet others in the same situation; a number of women maintained those connections, supporting each other after the course had ended.

*“At the “project”, they understood me … I found a family with the people from the project and I liked that a lot. But I couldn’t have done it differently because I felt comfortable with those women* [61].” “*A lot of the meetings was about making friends, I’ve got a busy life, now if I feel I’m not good at anything we can just ring each other* [37].” “*It was great being around women who were in a similar situation to me and I liked being able to talk about my own experience* [58].” “*… the small and large group discussions … provided an opportunity to connect and relate to other women in the programme* [58].”

##### Sharing

Women valued the opportunity to talk about their own emotions and problems and listen to others. They were able to do so because the class provided a non-judgemental, safe place. “Sharing” facilitated a sense of normality to their experiences.

*“Listening to them made us feel important … that was important … because like I always say, some people may be in the same situation as me* [61].” *“Sharing experiences and realising that they were not alone in what they felt (especially when these were negative feelings) helped women to normalise their experiences; in this way the course acted as a normalising catalyst* [38].” *“Being able to talk to someone and listen to advice* [61].” *“I learned that this is a shared human experience, and I’m not the only one who suffers* [58].”

##### Understanding

Women developed a greater understanding of themselves, their emotional state and perinatal mental health as a whole, as a result of attending the courses. In addition, they were able to understand what their needs were and when they most needed help.

*“I have learned how to better understand my thoughts and my body. How my thoughts can trigger feelings and how these thoughts are not always factual* [58].” *“I became aware of my mood and I realised that what I had was not something bad … what I really had was low self-esteem* [61].” *“Yeah, that was fine, it was good, learning things, you know, social support, turning it down, asking for help …* [37].”

##### Re-Adjusting and Normalising

Data suggest that for some women, the realisation that their experiences were not very different from others, combined with feeling less alone, facilitated an overall improvement in sense of emotional wellbeing. Women seemed to readjust how they viewed themselves, becoming kinder to themselves, and more accepting and comfortable about their thoughts, feelings and behaviours. For some women this process of re adjustment helped to “normalise” their depressive experience in relation to the context of others.

*“This is something that happens to women, after being involved in the project, it became more real to me* [61].” *“Being more accepting of myself, being more gentle to oneself, appreciating self-kindness, accepting anxiety as part of who I am, accepting my thoughts* [58].” *“Whenever I feel sad or depressed, I try to think about something nice, something that makes me feel happy, I learned all that there* [61].”

## 4. Discussion

### 4.1. Interventions

Over the last decade and a half research into antenatal group interventions to improve maternal psychological well-being has increased exponentially. The studies which met inclusion criteria for this review investigated interventions based on three psychological approaches: CBT, IPT and mindfulness. These three approaches are based on clear theoretical models and there is some evidence of their efficacy in improving psychological well-being generally and, particularly for CBT and IPT, during the perinatal period [22,24,25,26,27].

### 4.2. Heterogeneity and Methodological Limitations

Heterogeneity in terms of interventions, participants and study characteristics made it difficult to compare outcomes, interventions and psychological approaches. The format and contents of interventions varied even within each approach, though there were several CBT- and IPT-based interventions which were based on the same programmes. Further heterogeneity comes from the varying formats of interventions and the international nature of the studies, which originate in seven countries, potentially providing very different contexts.

Of particular importance is the lack of comparability with respect to the screening process; women were screened at different times and in various different ways, as demonstrated by the variation in the proportion of the population which screened positive. The differences in what outcomes were measured, when they were measured and how they were measured were also considerable. Outcome measures in particular were not uniform, therefore precluding direct comparisons. As a result, it is difficult to compare individual interventions and draw conclusions about the efficacy of psychological approaches.

Methodological limitations were common, particularly in terms of attrition, small sample sizes, unclear randomisation, differences between those who attended interventions and/or completed data collection and those who did not, and in sufficient analysis and presentation of results.

### 4.3. Effectiveness of Interventions

It is striking that even though almost all studies measured depressive symptoms and/or depression prevalence, most found no evidence of efficacy. Two studies found some evidence of a reduction in levels of depressive symptoms after CBT-based interventions, but both need to be treated with caution due to high attrition rates and low numbers. The evidence for a reduction in the prevalence of depression is also not very robust in the case of two IPT-based interventions [38,55], while two further, more methodologically robust, studies [40,42] provide more convincing evidence that the interventions in question, CBT/IPT-based and CBT-based respectively, may reduce the incidence of depression. Two studies which measured cortisol levels to assess the effect of CBT-based interventions on stress reduction [36,45] provide some evidence for the efficacy of these interventions in the reduction of stress. However, it is important to consider that the link between salivary cortisol is an indirect measure of stress and is moderate by other psychological and biological variables [64]; evidence for the link between measurement of cortisol levels and perceived stress is ambiguous [65].

Except for baseline psychological status, participant characteristics did not seem to impact on results. It is conceivable that the gestational age at which women took part in the intervention may affect outcomes. As research [66,67] suggests that maternal stress and anxiety have a changing course across trimesters and after birth, the efficacy of interventions may vary. However, most studies did not provide sufficient detail and the available data does not suggest a discernible pattern. For example, in two of the largest studies, which also show some evidence of a positive effect on depressive symptoms and depression prevalence [35,40], gestational ages are relatively low (mean 16 weeks) and relatively high (over 25 weeks), respectively.

A previous review of individual and group antenatal interventions for high-risk women [68] suggests that interventions which address interpersonal difficulties may be more effective. This applies to some of the effective interventions included in this review [40,41,44,60,62] but not others [28,35,42,46]. As the evidence base here is unconvincing, further research should explore the importance of including a specific focus on relationships and support. There is some evidence that individual interventions may be more effective [69], but differences between group and individual interventions need to be explored in more detail, particularly in the context of variability in the content and format of interventions.

It is impossible to draw overall conclusions about the efficacy of group interventions using a theoretical psychological framework. How well an intervention works appears to depend to a large extent on the individual design and delivery of the intervention and on the target participants. There is some evidence that antenatal group interventions may be more effective for women with higher baseline symptoms [35,40,41,42], supporting findings from a previous review [68]. However, not all studies compared women with higher and lower symptoms and differences in screening procedures and measures used make comparisons across studies difficult. It is therefore feasible that this increased effectiveness is due to a larger scope for improvement for women with higher symptoms at baseline. This is an area which would benefit from further investigation.

### 4.4. Women’s Experiences

#### 4.4.1. Qualitative Studies

Despite the lack of consistency in the quantitative literature, women’s positive experiences of these interventions are evidenced in the qualitative studies. Group-based sessions appear to be a platform through which pregnant women can make important connections with others. Data suggest that women considered “connectivity” particularly important in terms of making friends with other participants. These peer connections were significantly valued by women and the value of such has been referred to in other work including a meta-ethnography on the role of peer support in the context of perinatal mental illness [21], which demonstrated that the search for a peer environment in which women with perinatal mental illness can be honest about how they feel, is an essential part of the search for understanding and validation.

Several UK Department of Health policy statements support the use of nonprofessional providers or peers in health care settings. According to Simoni *et al.* (2011) [70], contemporary peer interventions derive from diverse conceptual and theoretical foundations that both guide and limit peer work. The peer principle is based on finding an affiliation with another, where life experience is similar, facilitating equality within the relationship [71]. Broader “peer” definitions draw on elements such as exchange of resources between individuals of equal status, similar adverse experiences, with key principles being founded on respect, shared responsibility, and mutual agreement [16,17,18,19,20].

For the women across these studies, an environment where women can be honest with each other, connect and share experiences, seemed to be instrumental in the process of normalization and improved emotional wellbeing. Based on this finding, we suggest that peer support networks have made a significant contribution to women positively experiencing these interventions. Despite these findings, the literature on peer-related health interventions remains largely atheoretical, and therefore it is difficult to comment on the precise mechanisms of, and to what extent, the peer support relationship has influenced women’s experiences of antenatal psychological group interventions, and ultimately influences outcomes as measured by traditional measures of anxiety, depression and stress.

#### 4.4.2. Women’s Feedback in Quantitative Studies

Participants’ feedback and evaluations within the quantitative studies were overwhelmingly positive. Several studies found that even though women were very satisfied with the intervention, there was no evidence of a reduction in depressive symptoms [42]. This suggests that either the studies fail to quantitatively measure the positive effects of the interventions or that women enjoy the interventions and gain some benefit from them, but that this benefit is not measurable or is about something that was not measured or is not traditionally assessed. It is noteworthy that many of the validated measures used in these studies have been critiqued and identified as lacking in both contemporary theoretical grounding and ecological validity, which might explain, at least in part, the discordance between women’s quantitative and qualitative experiences [72].

### 4.5. Combining Qualitative and Quantitaive

Findings from the thematic synthesis illuminate the quantitative data to some extent, providing an exploratory account of the consequences of engagement with antenatal psychological group interventions for women. Our mixed method framework offers an innovative approach to this review, and allows us to provide some further comments about the impact of these types of interventions. Overall, the impacts upon depressive symptoms, depressive episodes, anxiety, and perceived stress, have been difficult to determine and comment upon due to the methodological weaknesses and the heterogeneity of the quantitative studies. However, the qualitative data are suggestive of some improvements in women’s wellbeing that seem to elude measurement, for example gaining confidence [38], finding enjoyment in the classes [37], feeling optimistic even in the face of adversity and stress [61], and being able to lower one’s own stress levels [58]. This is important given that it is impossible to draw overall conclusions about the interventions’ efficacy. Gaining greater insights into the differing level of difficulties that these interventions may help to alleviate, may underpin more effective decisions about appropriate interventions for women. Thus, in this sense, increased understanding of “what works”, “for whom” and “why”, would appear to be a next step in terms of research and evidence in this aspect of perinatal mental health.

### 4.6. Strengths and Limitations of This Review

The limitations of this review depend to a large extent on the quality of the studies which were included. Unfortunately many studies had considerable methodological limitations, including small sample sizes, lack of robust randomisation and concealment, unclear baseline characteristics and inadequate presentation and analysis of findings; only one study included an active control group. Being in a group may have had an effect on participants’ psychological experiences by providing an element of peer support and shared experiences, as well as additional contact with a professional; peer support has been found to be effective in improving maternal psychological well-being [21,73]. Attrition rates were high for several studies and interventions and/or participant characteristics were not always adequately described. These limitations, which have been noted by other reviews [68,74,75], necessarily affect the extent to which this review is able to draw conclusions regarding the efficacy of interventions.

Despite these limitations, this systematic review has a valuable contribution to make, as it is the first review to focus on psychological group interventions in pregnancy targeting women with impaired psychological well-being and provides a review of quantitative as well as qualitative evidence. It provides an overview of the types of interventions and psychological approaches taken and found that only a few studies had a measurable significant beneficial effect, as well as some evidence that women with high baseline symptoms may benefit more.

## 5. Conclusions

This systematic review included 19 studies evaluating antenatal group interventions based on CBT, IPT and mindfulness. While there was some evidence of the efficacy of some interventions, mostly those based on CBT and IPT, the overall evidence was weak. Methodological limitations and the diversity in interventions, participants, screening procedures and outcome measures made it difficult to compare interventions and draw definite conclusions. Attrition was a considerable problem for the CBT-based interventions; in many studies only a relatively small proportion of participants attended the majority of sessions and/or completed data collection. There is some evidence that women with higher baseline psychological symptoms may benefit more from antenatal group interventions; further research in this area is needed. A meta-ethnography of the qualitative papers suggests that women who participated in the interventions valued them as an opportunity to make connections with others, gain peer support, normalise their feelings and improve their sense of well-being. Feedback and evaluations by participants in the quantitative studies were also largely positive; women said they had benefitted from interventions even if there was no quantitative evidence of efficacy. This suggests a need for further exploration of women’s experiences of antenatal interventions, as well as consideration of what are appropriate outcomes and effective measurements, perhaps using multiple outcome measures.

## Figures and Tables

**Table 1:** Included studies: interventions, participants and study design.

Intervention	Participants	Study Design
Quantitative Studies		
**Austin *et al.* 2008 (Australia) [23]**		
Brief CBT intervention *Aims:* preventing/managing stress, anxiety and low mood *Length*: 12 h (6 × 2 h) *Group size*: no information *Additional*: information booklet, follow-up session *Facilitator*: clinical psychologist, midwife *Evaluation/monitoring*: no information	277 women with depression/anxiety symptoms or at risk of depression/anxiety *Characteristics*: mean age 31 years; 97.8% partnered; 9.7% low income; 88.1% English speaking; 65.3% first child *Mean gestational age*: 25.7 weeks (range: 14 to 36 weeks) *Baseline symptoms*: MINI: depression 19.1%, anxiety 26%, depression or anxiety 32.5%; EPDS > 12 14.3% of completers	RCT; control: information booklet *Screening*: EPDS > 10/ANRQ > 23/history of depression ^1^ *Allocation*: randomisation on 2:1 basis (intervention:control) after screening; concealment unclear *Outcome measures*: depression (MINI), depressive symptoms (EPDS), anxiety (STAI, MINI) *Time points*: pre-/post-intervention; 2 and 4 months postnatal *Attrition/participation ^2^*: high attrition, low participation *Quality assessment*: B (differences in EPDS baseline scores; high rate attrition; wide range of gestational ages) Findings ^3^: No significant difference between groups (trend towards greater reduction in anxiety for intervention)
**Bittner *et al.*** **2014 (Germany)** [35]		
“LOS—Lebensfroh and optimistisch durch die Schwangerschaft“; CBT-based *Aims:* increased awareness of ongoing emotions, thoughts and behaviours *Length*: 12 h (8 × 90 min) *Group size*: 4–6 *Factilitator*: clinical psychologist *Evaluation/monitoring*: participant feedback	160 women with elevated depressive/anxiety symptoms ^4^ *Characteristics*: mean age 29.5 years; 100% partnered; medium/high socio-economic status; 64.9% first child *Mean gestational age*: 16.4 weeks *Baseline symptoms*: STAI 38; EPDS: 7.4 (int), 5.9 (con)	RCT; control: usual care *Screening*: PDQ > 14 / STAI > 36 / BDI-S > 20 *Allocation*: random; concealed *Outcome measures*: depressive symptoms (EPDS), anxiety (STAI), dysfunctional attitudes (DAS), anxiety sensitivity (ASI), social support (SOZU), quality of relationship (PFB), fear of childbirth (GAS) *Time points*: pre-/post-intervention; 3 months postnatal *Attrition/participation*: high attrition, low participation *Quality assessment*: A (but high drop-out rate) Findings: no intervention effect on anxiety or depression; positive short-term effect on quality of relationship for intervention; *women with elevated baseline depressive symptoms*: significant decrease in depressive symptoms postnatally in intervention group
**Brugha *et al.* 2000 (UK) [39]**		
“Preparing for Parenthood“; CBT elements and psychoeducation *Aims:* Preventing PND *Length*: 12 h (6 × 2 h) *Group size*: 8–16 *Additional*: introductory meeting, PN reunion; one session with partners *Facilitator*: nurses, occupational therapists *Evaluation/montitoring*: participant feedback; supervision	209 women at increased risk of depression *Characteristics*: median age 19 years; 73% European, others mostly Asian; 100% first child *Gestational age*: >28 weeks *Baseline symptoms*: GHQ-D high: 22/23%	RCT; control: usual care *Screening*: “Pregnancy and You“ screening questionnaire ^5^; 12–20 weeks *Allocation*: randomisation after screening (stratification based on 3 diagnostic factors); concealed *Outcome measures*: depression (SCAN), depressive symptoms (modified GHQ-D, EPDS); various risk factors for PND *Time points*: pre-intervention; 3 months postnatal *Attrition/participation*: low attrition, low participation *Quality assessment*: B (high attrition rates, insufficient detail on baseline comparisons) Findings: no intervention effect on levels of postnatal depression
**Crockett *et al.* 2008 (US)** [59]		
“Reach Out, Stand Strong: Essentials for New Moms“ (ROSE) Program; IPT-based *Aims*: preventing PND *Length*: 6 h (4 × 90 min) *Group size*: no information *Additional*: individual PN booster session *Facilitator*: counsellors *Evaluation/monitoring*: participant feedback; audiotaping for adherence/supervision	36 low-income African-American women, at risk of PND *Characteristics*: mean age 23.4 years; 13.9% partnered; mostly rural, low-income; 100% African American; 61.1% first child *Gestational age range*: 24–31 weeks *Baseline symptoms*: CSQ: mean score 34.5 ^6^, no reported previous depressive episodes	Pilot RCT; control: usual care *Screening*: CSQ ≥ 27 *Allocation*: randomisation after screening; no further information *Outcome measures*: depressive symptoms (EPDS), PN adjustment (PPAQ), parenting stress (PSI) *Time points*: pre-/post-intervention; 2–3 weeks and 3 months postnatally *Attrition/participation*: medium attrition, high participation *Quality assessment*: B (no information on allocation; results not presented comprehensively, small sample) Findings: significant increase in PN adjustment for intervention; no difference between groups in EPDS scores
**Guardino *et al.* 2014 (US) [28]**		
Mindful Awareness Practices (MAPS) course at UCLA Semel Institute’s (ongoing course, not specifically for pregnancy) *Aims:* Reducing stress *Length*: 12 h (6 × 2 h) *Group size*: no information *Facilitator*: no information *Evaluation/monitoring*: no information	47 women with raised stress and/or anxiety *Characteristics*: mean age 33.1 years; 93.5% partnered; medium-high socio-economic status; 66% white; 78% first child *Gestational age range*: 10–25 weeks *Baseline symptoms*: previous diagnosis of depression 30%; previous diagnosis of anxiety disorder 31%; STAI 45.7, PSS 41.8	Pilot RCT; control: reading (pregnancy book) *Screening*: PSS > 34 / PSA > 11 *Allocation*: randomisation (computerised) after screening; concealment unclear *Outcome measures*: perceived stress (PSS), pregnancy-specific anxiety (PSA), pregnancy-related anxiety (PRA), anxiety (STAI), mindfulness (FFMQ) *Time points*: pre-/post-intervention; 6 weeks after intervention *Attrition/participation*: medium attrition, medium participation *Quality assessment*: C (small sample; wide range of gestational ages; follow-up for some postnatally, others are possibly still pregnant) Findings: significantly larger decreases in pregnancy-specific anxiety pre- to post-intervention than control, not sustained at 6 weeks post-intervention
**Kozinszky *et al.* 2012 (Hungary) [40]**		
CBT and IPT elements; including partners *Aims*: preventing PND *Length*: 12 h (4 × 3 h) *Group size*: <15 *Facilitator*: psychiatrists and health visitors *Evaluation/monitoring*: sessions recorded randomly checked for adherence	1719 women *Characteristics*: mean age 27 years; 66% partnered; 14% low-income; 61% first child *Gestational age*: >25 weeks *Baseline symptoms*: antenatal depression 18.4%; history of major depression 5.6%	RCT; control: routine antenatal course *Screening*: none (but subgroup analysis) *Allocation*: randomisation (computerised) after eligibility criteria met, on ¾:1 (intervention:control) basis; concealment unclear *Outcome measures*: depression (LQ > 11), depressive symptoms (LQ); questionnaire on various risk factors of PND *Time points*: pre-intervention; 6-8 weeks postnatal *Attrition/participation*: low attrition, no information on participation *Quality assessment*: A (large sample; length of follow-up relatively short; no information on attendance rates) Findings: significantly reduced risk of PND and depressive symptoms in intervention group; *women with AN depression:* 17.8% risk reduction (without AND: 0.4%); intervention: significantly lower perceived lack of support from partner
**Lara *et al.* 2010 (Mexico)** [42]		
“Salud Mental de Mamás y Bebés“; CBT-based *Aims*: preventing PND *Length*: 16 h (8 × 2 h) *Group size*: 5–10 *Additional*: 2 PN individual booster sessions; self-help book on depression (also for control) *Facilitator*: no information *Evaluation/monitoring*: participant feedback; sessions filmed and reviewed	377 women at high risk of depression *Characteristics*: mean age: 26.9 years; 85.7% partnered; low-middle socio-economic status; 25.5% first child *Mean gestational age*: 26.9 weeks *Baseline symptoms*: major depression (SCID) 17.4%, BDI-II ≥ 14 62.7%, anxiety (SCL-90) 14.8%	RCT; control: usual care *Screening*: CES-D ≥ 16 / self-reported history of depression ^7^ *Allocation*: randomisation (block), before or after screening (see “quality assessment“); concealment unclear *Outcome measures*: depression (SCID), depressive symptoms (BDI-II), anxiety (SCL-90) *Time points*: baseline; 6 weeks and 4–6 months postnatal *Attrition/participation*: high attrition, low participation *Quality assessment*: B (randomisation problematic: for 44% baseline interview took place before randomisation, for others after, resulting in significant differences in depressive symptoms: women who knew which group they had been randomised to reported higher CES-D scores; high attrition rate before start of intervention) Findings: significantly lower cumulative incidence of (new) major depression over all time points for intervention group; no intervention effect on depressive symptoms
**Le *et al.* 2011 (US)** [60]		
“Mamás y Bebés / Mothers and Babies Course“; cognitive behavioural stress managrement *Aims*: preventing depression *Format*: 16 h (8 × 2 h) *Group size*: no information *Additional*: 3 individual PN booster sessions *Facilitator*: researchers *Evaluation/monitoring*: sessions filmed and selectively reviewed; supervision	217 low-income women at high risk of depression *Characteristics*: mean age 25 years; 69.6% (int) / 57.1% (con) partnered; mostly low-income; mostly Central and South American immigrants; 38.4% (int) / 46.7% (con) first child *Mean gestational age*: ≤24 weeks at baseline *Baseline symptoms*: BDI-II 15.7 int, 14.9 con, BDI-II ≥ 20 25% int, 24% con	RCT; control: usual care *Screening*: CES-D ≥ 16 / self-reported personal or family history *Allocation*: randomization (sealed envelope) after screening; concealed *Outcome measures*: depressive symptoms (BDI-II), major depressive episodes (MS) *Time points*: pre-/post-intervention; 6 weeks, 4 and 12 months PN *Attrition/participation*: high attrition, low participation *Quality assessment*: C (low participation and high attrition; baseline comparatibility of groups problematic) Findings: significantly fewer depressive symptoms immediately post-intervention (small effect size); fewer cases of moderate depression (BDI-II ≥ 20) post-intervention; stronger size of effects for women who attended more session; no difference postnatally; no difference in cumulative incidence of major depressive episodes
**Leung & Lam 2012 (China, Hong Kong) [41]**		
IPT-based *Aims*: reducing stress and depressive symptoms, enhancing happiness and self-efficacy in managing conflict *Length*: 6 h (4 × 90 min) *Group size*: no information *Facilitator*: no information *Evaluation/monitoring*: sessions video taped and reviewed	156 women *Characteristics*: mean age 31.2 years; 91.8% partnered; 73.5% first child *Mean gestational age*: 20 weeks *Baseline symptoms*: EPDS > 12 41% (int) / 30% (con)	Multisite RCT; control: usual care *Screening*: none (but subgroup analysis) *Allocation*: permuted block randomisation (sub-sets of four) after eligibility established; concealed *Outcome measures*: stress (PSS), depressive symptoms (EPDS), happiness (SHS), self-efficacy in managing conflict (REM), perceived ability to cooperate (single question), perceived health (single question) *Time points*: pre-/post-intervention; 6–8 weeks postnatal *Attrition/participation*: low attrition, high participation *Quality assessment*: A (but intervention very culturally specific, focus on relationship with grandparents) Findings: significantly lower perceived stress (moderate effect size) and smaller decrease in happiness (small/moderate effect size) post-intervention, not sustained postnatally; *women with depressive baseline symptoms:* lower stress (moderate effect size) and smaller decrease in happiness post-intervention, increased relationship self-efficacy (large effect size); no difference in depressive symptoms
**Muñoz *et al.* 2007 (US) [43]**		
“Mamás y Bebés / Mothers and Babies Course“; cognitive behavioural stress managrement *Aims*: preventing depression *Length*: 24 h (12 × 2 h) *Group size*: 3–8 *Additional*: 4 PN booster sessions *Facilitator*: researchers *Evaluation/monitoring*: sessions filmed and reviewed, supervision	41 women at high risk of depression *Characteristics*: mean age 24.9 years; 71.4% (int) / 80% (con) partnered; mostly low-income; 70% born in Mexico and Central America *Mean gestational age*: 16.1 (int) / 15.7 (con) weeks *Baseline symptoms*: CES-D 16.0 (int) / 16.8 (con); history of MDE 47.6% (int) / 60% (con)	RCT; control: usual care *Screening*: CES-D ≥ 16 / past history of major depressive episode (MMS) *Allocation*: randomisation after screening; method and concealment unclear *Outcome measures*: depression (MMS), depressive symptoms (CES-D, EPDS) *Time points*: pre-/post-intervention; 1, 3, 6 and 12 months postnatal *Attrition/participation*: low attrition, low participation *Quality assessment*: B (no information on randomisation, relatively low attendance, no analysis of attenders/non-attenders; postnatal booster sessions may affect outcomes) Findings: no significant differences in depressive symptoms or incidence of MDEs
**Richter *et al.* 2012 (Germany) [36]**		
*See Bittner et al. 2014* [35]	129 women with elevated stress, anxiety or depression ^5^ *See Bittner et al. 2014* [35] *for further details*	RCT; control: usual care *Screening*: PDQ > 14 / STAI > 36 / BDI-S > 20; 10–15 weeks *Allocation*: random (random allocation sequences); concealed *Outcome measures*: stress (PDQ, PSS), salivary cortisol *Time points*: pre-/post-intervention; 3 months postnatal *Attrition/participation*: high attrition, low participation *Quality assessment*: A (but high attrition) Findings: intervention: significant change in morning cortisol compared to control post-intervention but not postnatally; no significant difference in perceived stress
**Urizar & Muñoz 2011 (US) [45]**		
*See Muñoz et al. 2007* [43]	86 women at-risk of depression *Characteristics*: mean age 25.4 years; >72% partnered; mostly low-income; >75% born in Mexico and Central America; mostly second or subsequent child *Mean gestational age*: 16–17 weeks (range: 6–27 weeks) *Baseline symptoms*: CES-D 20.6 (int), 23.7 (con1), 9.4 (con2); history of MDE 33.3% (int), 66.% (con1), 0% (con2)	RCT; control 1: usual care, control 2: low risk *Screening*: CES-D ≥ 16 / past history of major depressive episode (MMS) *Allocation*: randomization after screening; method and concealment *Outcome measures*: salivary cortisol levels (mother & infant), perceived stress, depressive symptoms (CES-D), depression (MMS), positive/negative affect (PANAS) *Time points*: baseline; 6 and 18 months postnatal *Attrition/participation*: low attrition, low participation *Quality assessment*: B (postnatal booster sessions may affect outcomes; large variation in gestational age) Findings: intervention and control 2: significantly lower infant cortisol levels at 6 months PN; intervention: lower maternal cortisol levels than control 1 at 18 months PN; significantly higher levels of perceived stress at 6 months PN in intervention group
**Vieten & Astin 2008 (US) [46]**		
“Mindful Motherhood“, based on Mindfulness-Based Stress Reduction *Aims*: reducing stress and improving mood *Length*: 16 h (8 × 2 h) *Group size*: no information *Additional*: CD with guided meditations *Facilitator*: clinical psychologist *Evaluation/monitoring*: no information	31 women, with “mood concerns“ *Characteristics*: mean age 33.9 years; 100% partnered; medium-high socio-economic status; 74% white *Mean gestational age*: 25 weeks *Baseline symptoms*: perceived stress 20.1 int, 17.1 con; state anxiety 43.8 int, 35.6 con; CES-D 20.4 int, 14.2 con	Pilot RCT; control: wait-list control (postnatal) *Screening*: positive response to “Have you had a history of mood concerns for which you sought some form of treatment…?“ *Allocation*: randomisation after screening; concealment not clear *Outcome measures*: perceived stress (PSS), positive/negative affect (PANAS), depressive symptoms (CES-D), anxiety (STAI), affect regulation (ARM), mindfulness (MAAS) *Time points*: pre-/post-intervention; 3 months post-intervention *Attrition/participation*: low attrition, high participation *Quality assessment*: C (no baseline comparisons between groups but there seem to be differences; no information on attenders / completers; follow-up at different times, postnatally or during pregnancy) Findings: significantly reduced state anxiety and negative affect with large effect sizes post-intervention compared to control, not sustained 3 months post-intervention
**Zlotnick *et al.* 2001 (US)** [61]		
“Survival Skills for New Moms“; IPT-based *Aims*: preventing PND *Length*: 4 h (4 × 1 h) *Group size*: no information *Facilitator*: no information *Evaluation/monitoring*: no information	37 women on public assistance at risk of PND *Characteristics*: mean age 23.4 years; 23% partnered; low-income; 45% Caucasian *Gestational age range*: 20–32 weeks *Baseline symptoms*: BDI > 10: 70% int, 44% con; history of depression: 70% int, 51% con	Pilot RCT; control: usual care *Screening*: at least 1 predictor for risk factors for PND ^8^ *Allocation*: randomisation after screening; method and concealment unclear *Outcome measures*: depressive symptoms (BDI), depression (SCID) *Time points*: pre-/post-intervention; 3 months postnatal *Attrition/participation*: low attrition, high participation *Quality assessment*: C (limited details; short intervention; apparent differences at baseline between intervention and control; small sample) Findings: Intervention: women significantly less likely to develop PND
**Zlotnick *et al.* 2006 (US)** [44]		
“Reach Out, Stand Strong: Essentials for New Moms“ (ROSE) Program; IPT-based *Aims*: preventing PND *Length*: 4 h (4 × 1 h) *Group size*: 3–5 *Additional*: individual PN booster *Facilitator*: nurses *Evaluation/monitoring*: no information	99 women at risk of PND *Characteristics*: mean age 22.4 years; 33.3% partnered; low-income; 44% Hispanic *Gestational age range*: 23–32 weeks *Baseline symptoms*: previous MDE 31.3%; BDI 15.6	RCT; control: usual care *Screening*: CSQ ≥ 27 *Allocation*: randomisation after screening (stratified for previous episode of depression); concealment unclear *Outcome measures*: depressive symptoms (BDI), depression (LIFE depression module), social adjustment (RIFT) *Time points*: pre-intervention; 3 months postnatal *Attrition/participation*: low attrition, medium participation *Quality assessment*: B (intervention relatively short, no measurement immediately after the intervention; no detailed description of the intervention) Findings: 3 months PN: fewer women in intervention with PND than in control (4% *vs*. 20%); no significant difference between groups for depression severity (BDI) or social adjustment at 3 months PN
**Qualitative Studies**		
**Goodman *et al.* 2014 (US) [58]**		
Coping with anxiety through Living Mindfully (CALM) Pregnancy; mindfulness-based cognitive therapy adapted for pregnant women with anxiety *Aims*: coping with anxiety *Format*: 16 h (8 × 2 h) *Group size*: 6–12 *Additional*: MP3s of meditations for home practice *Facilitator*: clinical social worker *Evaluation/monitoring*: audiotaped and reviewed for fidelity and supervision	24 women with elevated anxiety symptoms and no greater than moderate levels of depression *Characteristics*: mean age 33.5 years; 96% partnered; 75% white/non-Hispanic; 66.6% first child *Mean gestational age*: 15.5; range: 6–27 weeks *Baseline symptoms*: 70.8% met criteria for GAD	Qualitative content analysis *Screening*: PSWQ ≥ 45 / GAD-7 ≥ 10 / BAI ≥ 11 / PHQ-9 < 15 *Data collection*: written response to open-ended questions *Attrition/participation*: low attrition, high participation *Quality assessment*: B (insufficient details of how study was explained to participants, insufficient details of data analysis; limited discussion regarding credibility of findings and value of research) Findings: seven categories: skill building; connection; universality; acceptance and self-kindness; decreased reactivity; cognitive changes; insight
**Le *et al.* 2013 (US)** [61]		
“Mamás y Bebés / Mothers and Babies Course“; cognitive behavioural stress managrement *Aims*: preventing depression *Format*: 16 h (8 × 2 h) *Group size*: no information *Additional*: 3 individual PN booster sessions *Facilitator*: researchers *Evaluation/monitoring*: sessions filmed and selectively viewed	39 women (participants in Le *et al.* 2011 [60]) *Characteristics*: mean age 27.8 years; 61.5% partnered; mostly Central and South American immigrants *Mean gestational age*: no information *Baseline symptoms*: CES-D ≥ 16 23.1%; personal history of depression 69.4%	Inductive comparative analysis *Screening*: CES-D ≥ 16 / past history of major depressive episode (MMS) *Data collection*: semi-structured interviews *Attrition/participation*: low attrition, low participation *Quality assessment*: B (lacking in detail about recruitment strategy, data collection, relationship between researcher and participants, data analysis) Findings: Women valued participating in the course; support network; awareness of mood; increased maternal efficacy; reduced isolation; child development; group experience; using tool
**Wheatley & Brugha 1999 (UK)** [37]		
*See Brugha et al. 2000* [39]; CBT-based	9 women (subsample of Brugha *et al.* 2000 [39]) *Characteristics*: mean age 25.6 years; 68.7% white; 100% first child *Gestational age*: >28 weeks *Baseline symptoms*: unable to determine	Grounded theory *Screening*: “Pregnancy and You“ screening questionnaire ^5^; 12–20 weeks *Data collection*: interviews *Attrition/participation*: low attrition, low participation *Quality assessment*: B (some detail lacking in how the study was explained to participants and data analysis; limited discussion regarding credibility of findings and value of research) Findings: themes: postnatal depression (lack of knowledge as protective or vulnerable); positive experience
**Wheatley *et al.* 2003 (UK) [38]**		
*See Brugha et al. 2000* [39]; *CBT-based*	*See Wheatley & Brugha 1999* [37]	Grounded theory (focus on engagement with intervention) *See Wheatley & Brugha 1999* [37] *Quality assessment*: B (no clear statement of the aim; lack of detail about aspects relating to the appropriateness of the recruitment strategy, data collection, consideration of the researcher/participant relationship, the ethical issues and data analysis) Findings: Themes: initial engagement (need for information about PND, PND taboo, decision-making, practicalities); maintaining engagement (sharing experiences, normalising, sensitivity of PND, positive experience, practicalities)

^1^ prior history of depression assessed on basis of ANRQ question (feeling miserable/depressed prior to this pregnancy, led to interfering with relationships or seeking professional help). ^2^ attrition refers to women who dropped out of data colletion; participation relates to completion of the intervention. ^3^ significant (*p* < 0.5) outcomes are shown in bold; only significant differences between intervention and control are reported, not pre-/post-intervention differences. ^4^ though most women were within the normal/healthy range. ^5^ based on General Health Questionnaire depression items (presence of any one of the six depression items indicating AND on a modified GHQ-D was strongest predictor of PND). ^6^ 27 considered cut-off for high-risk status for PND. ^7^ based on question about history of depression (experienced feeling sad, lonely, not wanting to do anything etc with such an intensity and duration that you would say that you were depressed?)—included in 43.2% of sample. ^8^ previous episode of depression or PND, mild-moderate levels of depressive symptoms, poor social support, a life stressor within last 6 months. int: intervention group; con: control group; PN: postnatal; MDE: major depressive epis. *Instruments*: ARM: Affect Regulation Measure; ASI: Anxiety Sensitivity Index; BAI: Beck Anxiety Inventory; BDI: Beck Depression Inventory; BDI-II: Beck Depression Inventory (second edition); BDI-S: Beck Depression Inventory-simplified version; CES-D: Centre for Epidemiologic Studies Depression scale; CSQ: Cooper Survey Questionnaire; DAS: Dysfunctional Attitudes Scale; EPDS: Edinburgh Postnatal Depression Scale; FFMQ: Five Factor Mindfulness Questionnaire; GAD7: Generalised Anxiety Disorder 7-item; GAS: Geburtsangstskala (Fear of Childbirth Scale, German); GHQ-D: General Health Questionnaire (depression subscale); LIFE: Longitudinal Interval Follow-Up Evaluation (depression module); LQ: Leverton Questionnaire; M-CIDI: Munich-Composite International Diagnostic Interview; MAAS: Mindful Attention Awareness Scale; MINI: Mini International Neuropsychiatric Interview (depression & anxiety components); MMS : Maternal Mood Screener; MS: Mood Screener; PANAS : Positive and Negative Affect Schedule; PDQ: Prenatal Distress Questionnaire; PSS : Perceived Stress Scale; PFB: Partnerschaftsfragebogen (Quality of a Marriage or Intimate Relationship Scale, German); PHQ-9: Patient Health Quesitonnaire-9; PRA: Pregnancy-Related Anxiety scale; PSA: Pregnancy-Specific Anxiety scale; PPAQ: Postpartum Adjustment Questionnaire; PSI: Parenting Stress Index; PSS: Perceived Stress Scale; PSWQ: Penn State Worry Questionnaire; REM: Relationship Efficacy Measure; RIFT: Range of Impaired Functioning Tool; SCID: Structured Clinical Interview; SCL-90: Symptom Check List 90; SHS: Subjective Happiness Scale; STAI: Spielberger State-Trait Anxiety Inventory.

## Abbreviations

The following abbreviations are used in this manuscript:

BDI	Beck Depression Inventory
CASP	Critical Appraisal Skills Programme
CBT	Cognitive Behavioural Therapy
CES-D	Centre for Epidemiologic Studies Depression scale
EPDS	Edinburgh Postnatal Depression Scale
IPT	Interpersonal Therapy
MFN	Mindfulness
NICE	National Institute for Health and Care Excellence
PMI	Perinatal Mental Illness
PND	Postnatal Depression
PSS	Perceived Stress Scale
STAI	Spielberger State-Trait Anxiety Inventory
